# Synbiotic (*Lactiplantibacillus pentosus* GSSK2 and isomalto-oligosaccharides) supplementation modulates pathophysiology and gut dysbiosis in experimental metabolic syndrome

**DOI:** 10.1038/s41598-021-00601-2

**Published:** 2021-11-01

**Authors:** Sakshi Khanna, Mahendra Bishnoi, Kanthi Kiran Kondepudi, Geeta Shukla

**Affiliations:** 1 Department of Microbiology, Basic Medical Sciences Block A, South Campus, Panjab University, Chandigarh, 160014 India; 2 Healthy Gut Research Group, Food & Nutrition Biotechnology Division, National Agri-Food Biotechnology Institute (NABI), S.A.S. Nagar, Punjab, 140306 India

**Keywords:** Microbiology, Medical research

## Abstract

Metabolic syndrome a lifestyle disease, where diet and gut microbiota play a prodigious role in its initiation and progression. Prophylactic bio-interventions employing probiotics and prebiotics offer an alternate nutritional approach towards attenuating its progression. The present study aimed to evaluate the protective efficacy of a novel synbiotic (*Lactiplantibacillus pentosus* GSSK2 + isomalto-oligosaccharides) in comparison to orlistat in an experimental model of metabolic syndrome. It was observed that supplementation of synbiotic for 12 weeks to Sprague Dawley rats fed with high fat diet (HFD), ameliorated the morphometric parameters i.e. weight gain, abdominal circumference, Lee’s index, BMI and visceral fat deposition along with significantly increased fecal Bacteroidetes to Firmicutes ratio, elevated population of *Lactobacillus spp., Akkermansia spp., Faecalibacterium spp., Roseburia spp.* and decreased *Enterobacteriaceae* compared with HFD animals. Additionally, synbiotic administration to HFD animals exhibited improved glucose clearance, lipid biomarkers, alleviated oxidative stress, prevented leaky gut phenotype, reduced serum lipopolysaccharides and modulated the inflammatory, lipid and glucose metabolism genes along with restored histomorphology of adipose tissue, colon and liver compared with HFD animals. Taken together, the study highlights the protective potential of synbiotic in comparison with its individual components in ameliorating HFD-induced metabolic complications.

## Introduction

Metabolic syndrome, which encompasses obesity, dyslipidemia, hyperglycemia and chronic inflammation in metabolic tissues has emerged as a public health challenge affecting about one quarter of the world’s population and the prevalence is predicted to escalate in developed, developing and under developed countries^1^. Inclination towards high-energy diet and decreased physical activity have led to the increased prevalence of metabolic syndrome globally, and is associated with several pathophysiological alterations such as weight gain, ectopic fat deposition, hyperlipidemia, insulin resistance and alterations in gut microbiota, referred as the “second genome”^2,3^. Moreover, gut dysbiosis associated with high calorie intake, leads to significant loss of microbial diversity, increased energy harvest, disruption of gut barrier integrity, low grade inflammation resulting into metabolic endotoxemia, production of reactive oxygen species and deregulation of genes involved in lipid, glucose metabolism and inflammation which play a prodigious role in the advancement of metabolic complications^4^. Therefore, development of gut microbiota targeted strategy employing probiotics, prebiotics and synbiotics, that could potentially re-establish the gut homeostasis is budding as the novel prophylactic biointervention for alleviating metabolic disorders.

Probiotics are ‘live microorganisms that when administered in adequate amounts, confer a health benefit on the host’ and the health promoting potentials include maintenance of gut homeostasis, alienating pathogens, enhancing the bioavailability of nutrients, stimulation and modulation of host immune system^5,6^. Due to their multifarious benefits, probiotics are currently the major focus of attention to be explored as potential biotherapeutics for the management of various gastrointestinal ailments, liver damage, cancers, inflammatory and metabolic disorders^7,8^. Prebiotics, are non-digestible food ingredients that selectively stimulate the growth or activity of beneficial microorganisms in the host^9^. Biofermentation of prebiotic fibres releases short chain fatty acids, which are the messengers of cross talk between gut microbiota and host thereby regulating intestinal inflammatory response and colon health^10^.

Synbiotic, combination of probiotic with prebiotic, has been found to have a synergistic effect on host health and various experimental studies have indicated the protective and stimulatory effect of synbiotics^11,12^. Recently, we have observed that oral supplementation of probiotic isolate *L. pentosus* GSSK2 ameliorated the adiposity parameters and various biochemical components of metabolic syndrome and the probiotic isolate was found to metabolize isomalto-oligosaccharides (IMOs), a prebiotic reported to possess protective effects in an array of ailments by modulating immune response, improving gut flora, regulating carbohydrate and lipid metabolism^13–15^. Though some studies have reported that synbiotics can help in alleviating obesity and related complications, but no information is available with reference to the combination of probiotic with IMOs as synbiotic in ameliorating diet induced metabolic syndrome. Therefore, the need of hour is to explore novel biointerventions that are safer yet effective for the management of metabolic syndrome to overcome the adverse effects of commonly prescribed weight loss drugs like orlistat^16^. Thus, the present study aimed to evaluate the protective efficacy of a novel synbiotic (*L. pentosus* GSSK2 + IMOs) in comparison to orlistat in experimental model of metabolic syndrome.

## Results

### Improved morphometric parameters and reduced adiposity

**Figure 1:**
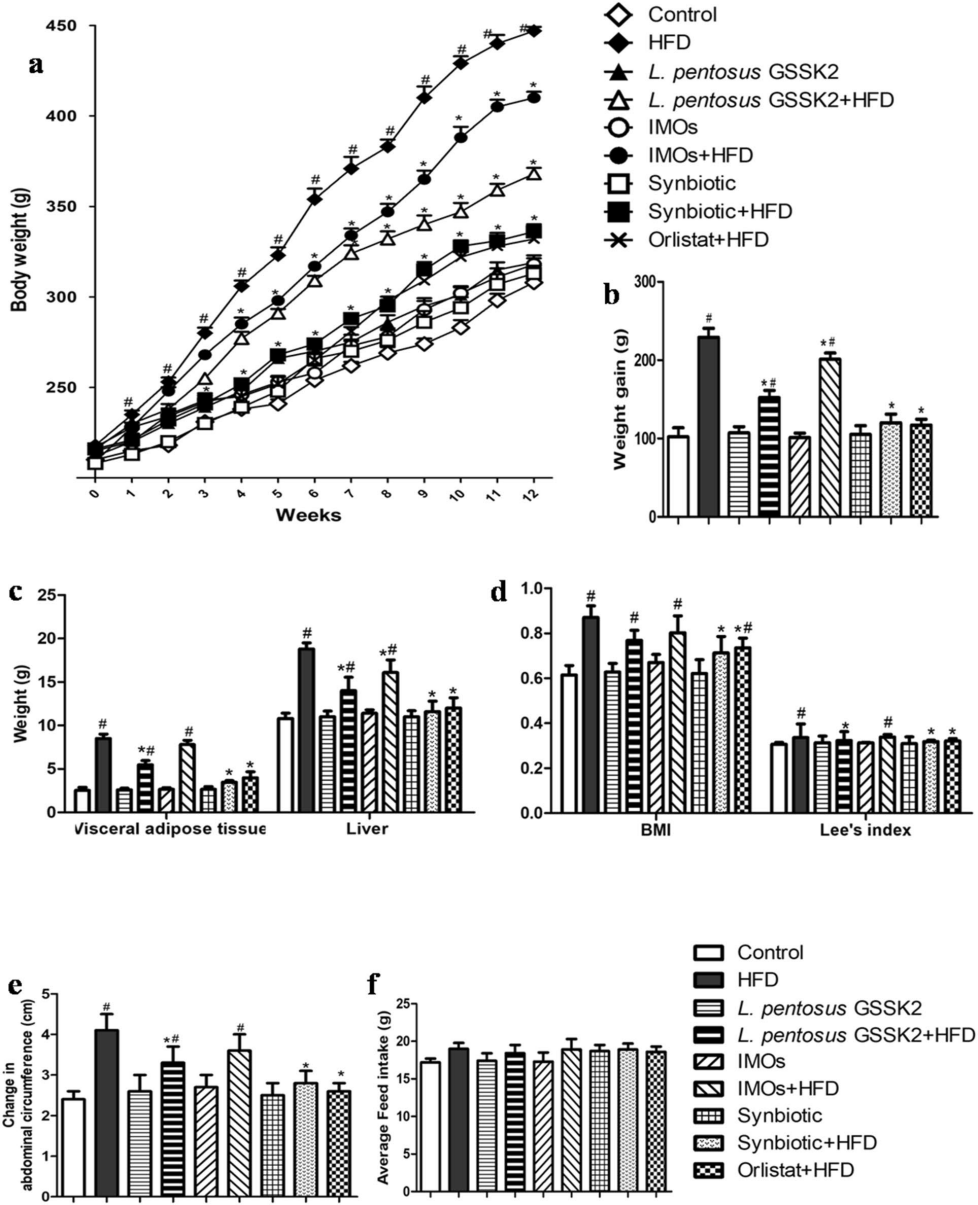
Morphometric parameters and adiposity markers in different groups of animals: (**a**) Body weight; (**b**) Weight gain; (**c**) Liver and adipose tissue weight; (**d**) BMI (g/cm^2^) and Lee’s index (**e**) Change in abdominal circumference; (**f**) Feed intake; Values are Mean ± SD, ^#^p < 0.05 versus control, *p < 0.05 versus HFD.

It was interesting to observe that animals fed either with synbiotic + HFD (Group VIII) or orlistat + HFD (Group IX) had significantly (p < 0.05) reduced body and liver weight, adipose tissue weight, abdominal circumference, BMI and Lee’s index followed by *L. pentosus* GSSK2 + HFD (Group IV) and IMOs + HFD (Group VI) compared with HFD (Group II) animals (Fig. 1a–e). However, animals belonging to either probiotic (Group III), prebiotic (Group V) or synbiotic (Group VII) had adiposity parameters comparable to control (Group I) and average feed intake was almost similar in animals belonging to all the groups (Group I–IX, Fig. 1f).

Interestingly, gross macroscopic examination of animals belonging to synbiotic + HFD (Group VIII) showed minimum fat deposits in adipose tissue, followed by orlistat + HFD (Group IX), *L. pentosus* GSSK2 + HFD (Group IV) and IMOs + HFD (Group VI) compared with HFD animals (Group II, Fig. S1 a to i).

### Improved glucose tolerance and reduced insulin

**Figure 2:**
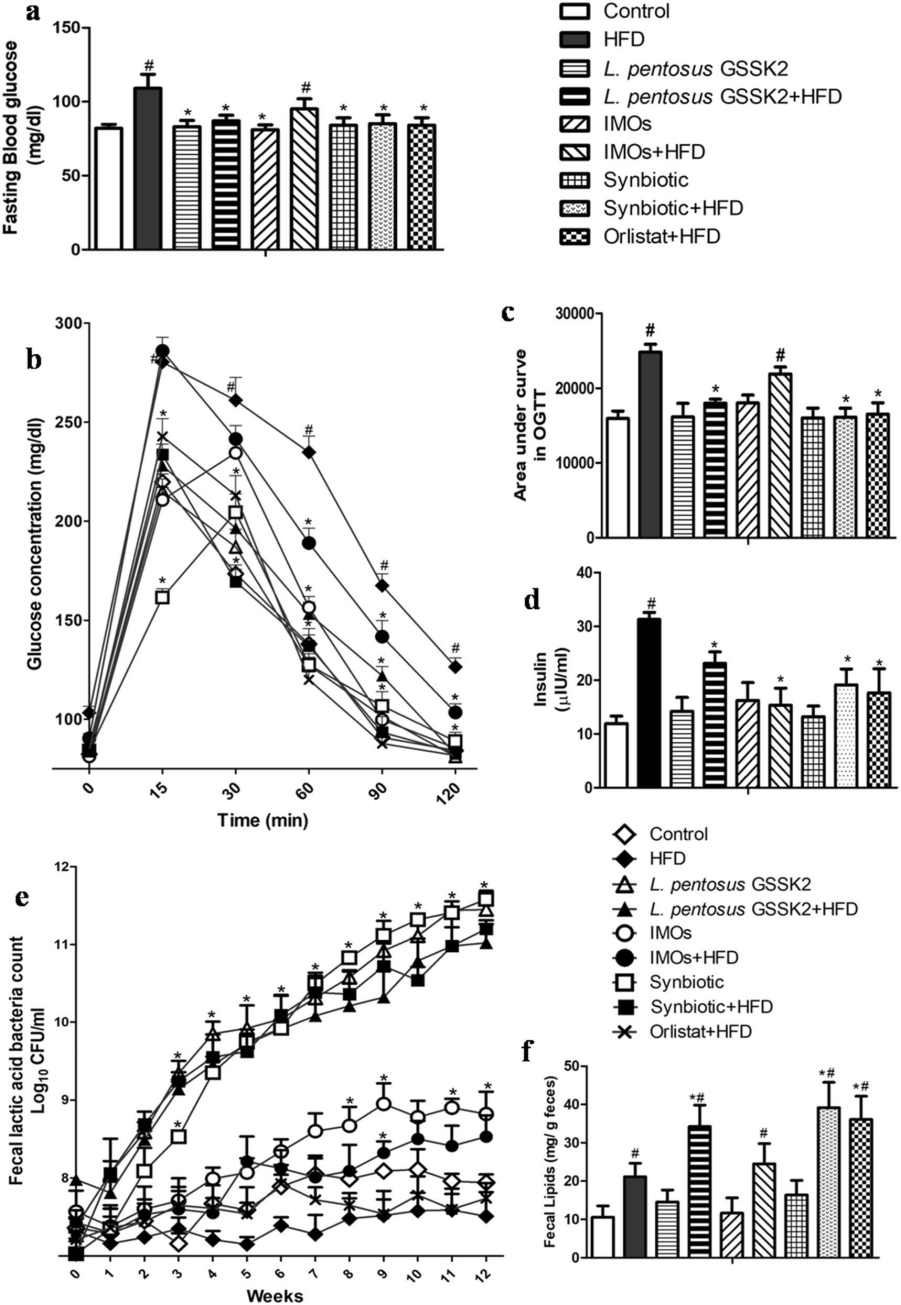
Effect of probiotic, prebiotic and synbiotic supplementation in animals belonging to various groups on: (**a**) Fasting blood glucose; (**b**) OGTT; (**c**) AUC of OGTT; (**d**) Blood insulin level; (**e**) Fecal lactic acid bacteria count (Log_10_ CFU/ml); (**f**) Fecal lipid excretion. Values are Mean ± SD, ^#^p < 0.05 versus control, *p < 0.05 versus HFD.

It was observed that administration of either probiotic *L. pentosus* GSSK2 (Group IV), synbiotic (Group VIII) or orlistat (Group IX) to HFD animals significantly (p < 0.05) lowered the fasting blood glucose level and enhanced glucose tolerance compared with HFD (Group II) animals that had increased fasting blood glucose level and impaired glucose clearance from circulation as depicted by an increase in the area under the concentration–time curve (AUC) during OGTT (Fig. 2a–c). However, animals belonging to IMOs + HFD (Group VI) did not show any significant improvement in glucose tolerance while animals supplemented either with probiotic (Group III), prebiotic (Group V) or synbiotic (Group VII) had blood glucose parameters comparable to control (Group I, Fig. 2a–c).Interestingly, serum insulin levels were significantly (p < 0.05) reduced in animals belonging to *L. pentosus* GSSK2 + HFD (Group IV), IMOs + HFD (Group VI) synbiotic + HFD (Group VIII) and orlistat + HFD (Group IX) animals compared with HFD (Group II) animals (Fig. 2d).

### Increased lactic acid bacteria (LAB) count and lipids in feces

The LAB count in feces, an indicator of healthy gut, increased significantly (p < 0.05) in animals belonging to synbiotic, probiotic and prebiotic inspite of HFD feeding compared with control (Group I) and HFD (Group II) animals but maximum increase in LAB was in synbiotic (Group VII) animals (Fig. 2e). Further, supplementation of synbiotic led to increased fecal lipid excretion inspite of HFD, compared with counter controls (Group II, IV, VI, IX) (Fig. 2f).

### Improved gut bacteria composition

**Figure 3:**
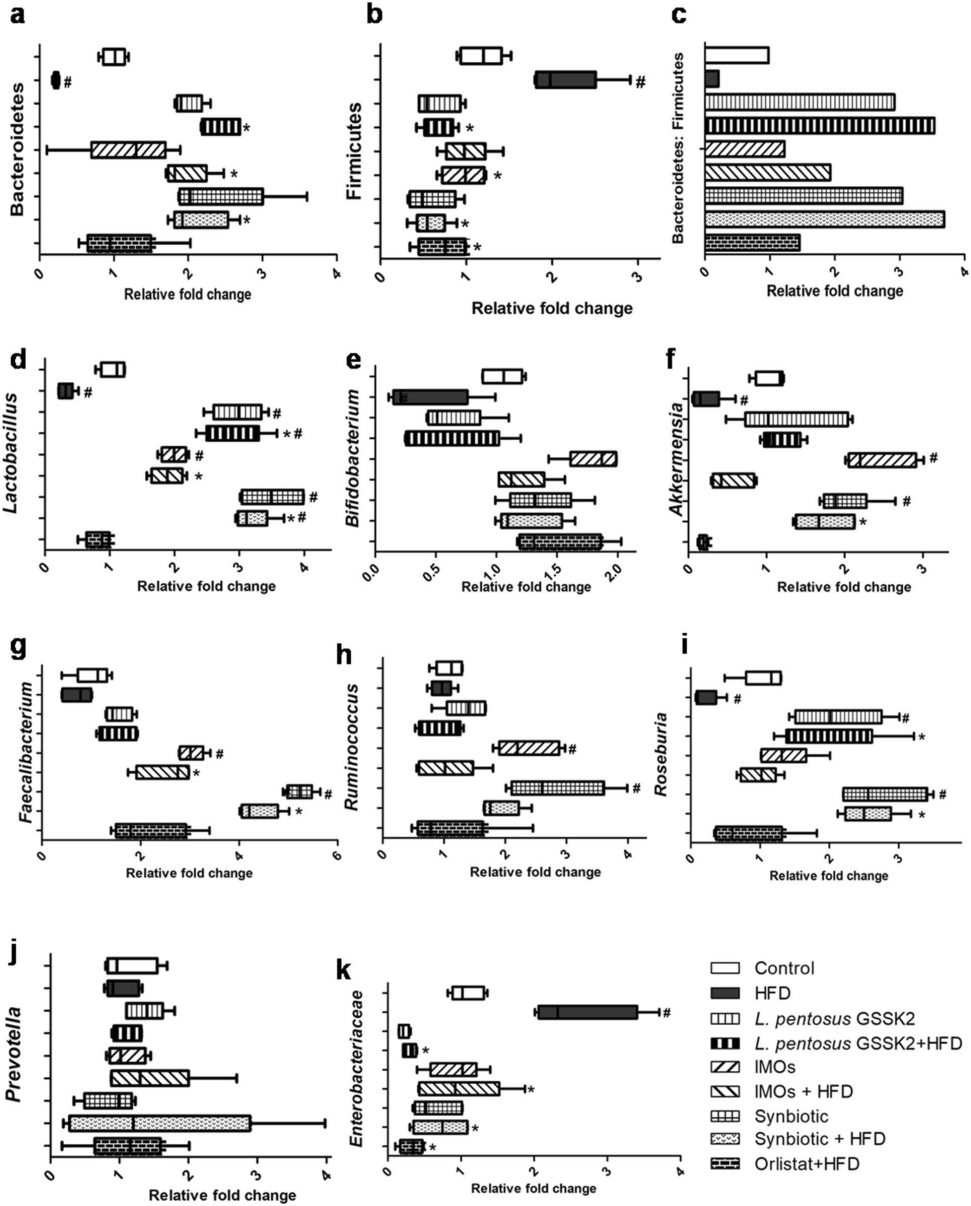
Relative bacterial abundance of different genera in animals belonging to different groups: (**a**) Bacteroidetes; (**b**) Firmicutes; (**c**) Bacteroidetes: Firmicutes; (**d**) *Lactobacillus;* (**e**) *Bifidobacterium;* (**f**) *Akkermansia; *(**g**)* Faecalibacterium;* (**h**) *Roseburia;* (**i**) *Ruminococcus*; (**j**) *Prevotella*; (**k**)* Enterobacteriaceae* by real-time PCR. Boxes represent the interquartile ranges, lines inside the boxes denote medians, ^#^p < 0.05 versus control, *p < 0.05 versus HFD.

Supplementation of synbiotic to HFD animals (Group VIII) led to maximum increase in the Bacteroidetes to Firmicutes ratio followed by *L. pentosus* GSSK2 + HFD (Group IV), IMOs + HFD (Group VI) and orlistat + HFD (Group IX) respectively, compared with HFD (Group II) animals (Fig. 3a–c). Further, animals belonging to synbiotic + HFD (Group VIII) had significantly (p < 0.05) elevated population of *Lactobacillus spp., Akkermansia spp., Faecalibacterium spp.,* and *Roseburia spp.* while *L. pentosus* GSSK2 + HFD (Group IV) had significantly (p < 0.05) high number of *Lactobacillus spp. and Roseburia spp.* while IMOs + HFD animals (Group VI) had increased population of *Lactobacillus spp.* and *Faecalibacterium spp* whereas, orlistat + HFD (Group IX) animals did not show any significant change in any of the bacterial genera compared with HFD animals (Fig. 3d–j). Moreover, *Enterobacteriaceae* population decreased significantly (p < 0.05) in synbiotic + HFD (Group VIII), *L. pentosus* GSSK2 + HFD (Group IV), IMOs + HFD (Group VI) and orlistat + HFD (Group IX) animals compared with HFD animals (Fig. 3k). Further, animals administered with probiotic (Group III) had increased abundance of *Lactobacillus spp.* and *Roseburia spp.,* prebiotic (Group V) animals had increased *Lactobacillus spp., Akkermansia spp, Faecalibacterium spp. and Ruminococcus spp.* while synbiotic (Group VII) animals had increased population of *Lactobacillus spp., Akkermansia spp, Faecalibacterium spp, Roseburia spp.,* and *Ruminococcus spp.* compared with control (Group I) (Fig. 3d–j).

### Improved serum biomarkers

It was found that animals belonging to synbiotic + HFD (Group VIII) and orlistat + HFD (Group IX) had significantly (p < 0.05) reduced obesity associated serum biomarkers i.e. total cholesterol, triglycerides and LDL cholesterol with increased HDL cholesterol while *L. pentosus* GSSK2 + HFD (Group IV) animals had significantly (p < 0.05) reduced levels of triglycerides and LDL-cholesterol and IMOs + HFD (Group VI) had reduced levels of triglycerides compared with HFD animals (Group II, Table 1).

**Table 1:** Serum biochemical parameters (lipid profile, liver function test, LPS, TNF-α and IL-6) of animals belonging to different groups.

Parameter	Groups								
	Control	HFD	*L. pentosus*	*L. pentosus* + HFD	IMOs	IMOs + HFD	Synbiotic	Synbiotic + HFD	Orlistat + HFD
Total cholesterol (mg/dl)	70.2 ± 9.5	110.8^#^ ± 9.9	75.6 ± 7.9	98.4^#^ ± 8.3	78 ± 9.0	93^#^ ± 10.4	71.4 ± 9.2	79.8* ± 9.6	82.6* ± 7.0
Triglycerides (mg/dl)	82 ± 5.6	118.4^#^ ± 11.7	76.8 ± 6.2	95.6* ± 5.2	79.8 ± 7.9	98.2* ± 9.6	85 ± 11.5	91* ± 9.2	89.2* ± 10.2
HDL cholesterol (mg/dl)	47.7 ± 4.4	32.1^#^ ± 4.3	43.2 ± 5.3	39.8 ± 3.6	43 ± 4.2	38 ± 5.1	45.2 ± 4.1	45* ± 7.1	45.4* ± 2.1
LDL cholesterol (mg/dl)	23.06 ± 3.4	40.72^#^ ± 2.7	28 ± 4.2	26.4* ± 6.5	26.8 ± 5.2	41.4^#^ ± 7.7	22.2 ± 8.2	25.3* ± 4.3	27.2* ± 5.5
SGOT (AST) (IU/L)	95.04 ± 9.8	160.8^#^ ± 9.9	100.6 ± 8.7	132.2* ± 8.0	104.4 ± 7.1	147^#^ ± 8.7	97.8 ± 4.3	106.6* ± 5.4	103.6* ± 8.1
SGPT (ALT) (IU/L)	62 ± 9.6	123.6^#^ ± 11.6	66.4 ± 6.6	80.8^#^* ± 7.9	79 ± 8.3	115^#^ ± 10.2	62 ± 6.4	73* ± 5.4	74.4* ± 6.1
Serum bilirubin (mg/dl)	0.48 ± 0.02	1.08^#^ ± 0.03	0.46 ± 0.02	0.66* ± 0.05	0.57 ± 0.04	0.72* ± 0.01	0.52 ± 0.03	0.58* ± 0.06	0.70* ± 0.04
Serum LPS (EU/L)	24.8 ± 3.02	108.5^#^ ± 16.3	20.6 ± 1.7	47.6* ± 6.9	27.3 ± 8.4	79.1^#^ ± 9.01	28.2 ± 4.3	38.9* ± 6.06	42.4* ± 8.04
Serum TNF-a (pg/ml)	2.3 ± 0.76	52.5^#^ ± 8.23	2.9 ± 0.59	30.3* ± 3.11	4.2 ± 0.78	41.1^#^ ± 9.01	3.6 ± 0.93	21.3* ± 1.42	14.7* ± 0.94
Serum IL-6 (ng/L)	1.9 ± 0.12	9.2^#^ ± 0.73	2.1 ± 0.51	4.3* ± 0.95	2.2 ± 0.14	4.5* ± 0.52	1.9 ± 0.15	3.4* ± 0.17	2.8* ± 0.24

Values are Mean ± SD, ^#^p < 0.05 versus control, *p < 0.05 versus HFD.

Further, *L. pentosus* GSSK2 + HFD (Group IV), synbiotic (Group VIII) and orlistat (Group IX) supplementation to HFD animals significantly (p < 0.05) reduced bilirubin, AST and ALT levels while IMOs + HFD (Group VI) animals showed significant reduction in bilirubin compared with HFD (Group II) animals (Table 1).

It was also observed that synbiotic + HFD (Group VIII) and orlistat (Group IX) animals had significantly (p < 0.05) reduced levels of serum LPS, TNF-α and IL-6 followed by *L. pentosus* GSSK2 + HFD (Group IV) and IMOs + HFD (Group VI) animals compared with their elevated levels in serum of HFD (Group II) animals (Table 1).

### Enhanced antioxidant and suppressed oxidant level

It was interesting to observe that supplementation of synbiotic to HFD animals (Group VIII) led to significant (p < 0.05) reduction in oxidant MDA level both in adipose tissue and colon whereas orlistat + HFD (Group IX) animals had maximum reduction of MDA in colon followed by *L. pentosus* GSSK2 + HFD (Group IV) and IMOs + HFD (Group VI) respectively compared with HFD animals (Table 2). Further, antioxidant GSH and SOD level increased in adipose tissue, colon as well as liver of synbiotic + HFD (Group VIII), followed by *L. pentosus* GSSK2 + HFD (Group IV) and orlistat + HFD (Group IX) while IMOs + HFD (Group VI) animals did not show significant (p < 0.05) change in GSH and SOD level compared with HFD animals (Table 2).

**Table 2:** Oxidant and antioxidant levels in animals belonging to different groups.

Groups	MDA(nM/mg protein)			GSH(nM/mg protein)			SOD(U/mg protein)		
	Adipose tissue	Colon	Liver	Adipose tissue	Colon	Liver	Adipose tissue	Colon	Liver
Control	46.6 ± 5.6	52.2 ± 4.3	58.3 ± 7.1	3.54 ± 0.2	2.72 ± 0.6	1.44 ± 0.1	3.12 ± 0.2	1.16 ± 0.1	1.25 ± 0.2
HFD	77.1^#^ ± 7.8	89.5^#^ ± 8.6	91.2^#^ ± 4.3	0.84^#^ ± 0.3	0.45^#^ ± 0.1	0.42^#^ ± 0.3	0.71^#^ ± 0.1	0.49^#^ ± 0.1	0.36^#^ ± 0.1
*L. pentosus*	42.2 ± 3.2	57* ± 3.6	52.2 ± 5.5	4.1 ± 0.31	2.51 ± 0.3	1.32 ± 0.6	3.01 ± 0.1	1.21 ± 0.3	0.98 ± 0.1
*L. pentosus* + HFD	65.2* ± 5.4	73.8 ± 4.3	63.8* ± 4.3	3.09 ± 0.1	2.03* ± 0.7	0.99* ± 0.4	2.6 ± 0.3	1.01* ± 0.4	0.66 ± 0.1
IMOs	50.4 ± 6.5	54.2* ± 4.6	61.2 ± 5.9	3.5 ± 0.3	2.10 ± 0.3	1.03 ± 0.5	3.13 ± 0.4	1.23 ± 0.2	1.04 ± 0.3
IMOs + HFD	71.2 ± 4.4	83.4 ± 5.3	88.3 ± 3.7	1.23 ± 0.9	0.99 ± 0.4	0.87 ± 0.4	1.45 ± 0.1	0.33 ± 0.3	0.78 ± 0.2
Synbiotic	48.2 ± 6.3	50.2 ± 4.4	60.6 ± 2.9	3.61 ± 0.8	2.54 ± 0.9	1.11 ± 0.2	2.98 ± 0.3	1.03 ± 0.7	1.34 ± 0.8
Synbiotic + HFD	55.5* ± 7.3	59.9* ± 6.4	61.2* ± 6.3	3.2* ± 0.7	1.99* ± 0.7	0.98* ± 0.3	2.12* ± 0.6	1.05* ± 0.2	0.99* ± 0.3
Orlistat + HFD	60.6* ± 4.3	55.5 ± 4.3	65.5* ± 4.4	3.01 ± 0.6	1.84* ± 0.1	0.52 ± 0.1	2.44* ± 0.4	1.09* ± 0.3	0.54 ± 0.5

Values are Mean ± SD, ^#^p < 0.05 versus control, *p < 0.05 versus HFD.

### Modulation of gene expression

**Figure 4:**
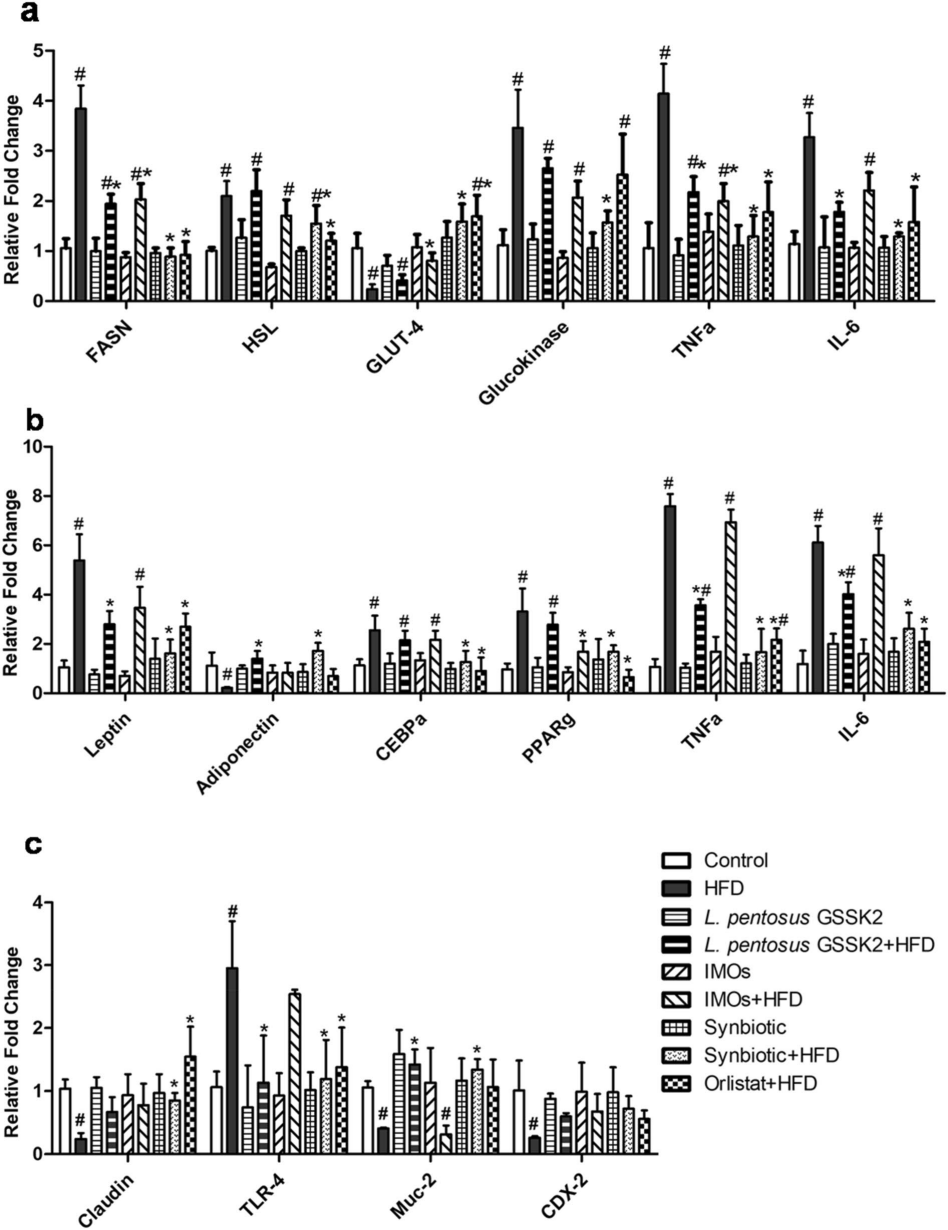
Relative gene expression of: (**a**) lipid metabolism genes, inflammatory markers, glucose metabolism genes in liver; (**b**) adiposity genes in adipose tissue; (**c**) gut integrity genes in colon of animals belonging to various groups by real-time PCR. Values are Mean ± SD, ^#^p < 0.05 versus control, *p < 0.05 versus HFD.

It was found that administration of synbiotic (Group VIII) and orlistat (Group IX) to HFD animals significantly (p < 0.05) downregulated the expression of lipid and glucose metabolism genes (FASN, HSL, GLUT-4 and glucokinase) and inflammatory markers (TNF-α and IL-6) in liver compared with HFD animals (Group II, Fig. 4a). *L. pentosus* GSSK2 + HFD (Group IV) animals had significantly (p < 0.05) decreased expression of FASN, TNF-α and IL-6 while FASN, TNF-α and GLUT-4 were downregulated in IMOs + HFD (Group VI) animals (Fig. 4a).

The expression of adiposity genes, C/EBPα and PPARγ in adipose tissue was significantly (p < 0.05) downregulated in orlistat + HFD (Group IX) followed by synbiotic + HFD (Group VIII), IMOs + HFD (Group VI) and *L. pentosus* GSSK2 + HFD (Group IV) respectively compared with HFD (Group II) while adiponectin expression was significantly (p < 0.05) upregulated in synbiotic + HFD (Group VIII) and *L. pentosus* GSSK2 + HFD (Group IV) compared with HFD (Group II) animals (Fig. 4b). Moreover, expression of adipokine gene i.e. leptin and inflammatory genes i.e. TNF-α and IL-6 was significantly (p < 0.05) downregulated in synbiotic + HFD (Group VIII) and orlistat + HFD (Group IX) followed by *L. pentosus* GSSK2 + HFD (Group IV) and IMOs + HFD (Group VI) compared to HFD (Group II, Fig. 4b).

The expression of gut integrity gene, claudin in colon was significantly (p < 0.05) upregulated in synbiotic + HFD (Group VIII) and orlistat + HFD (Group IX) while Muc-2 expression was upregulated in *L. pentosus* GSSK2 + HFD (Group IV) and synbiotic + HFD (Group VIII) compared with HFD (Group II) animals. Further, synbiotic + HFD animals (Group VIII) had maximum downregulation of TLR-4 expression followed by *L. pentosus* GSSK2 + HFD (Group IV), orlistat + HFD (Group IX) and IMOs + HFD (Group VI) animals respectively while there was no significant change in CDX-2 expression in animals belonging to various groups compared with HFD (Group II) animals (Fig. 4c).

### Histological modulation

**Figure 5:**
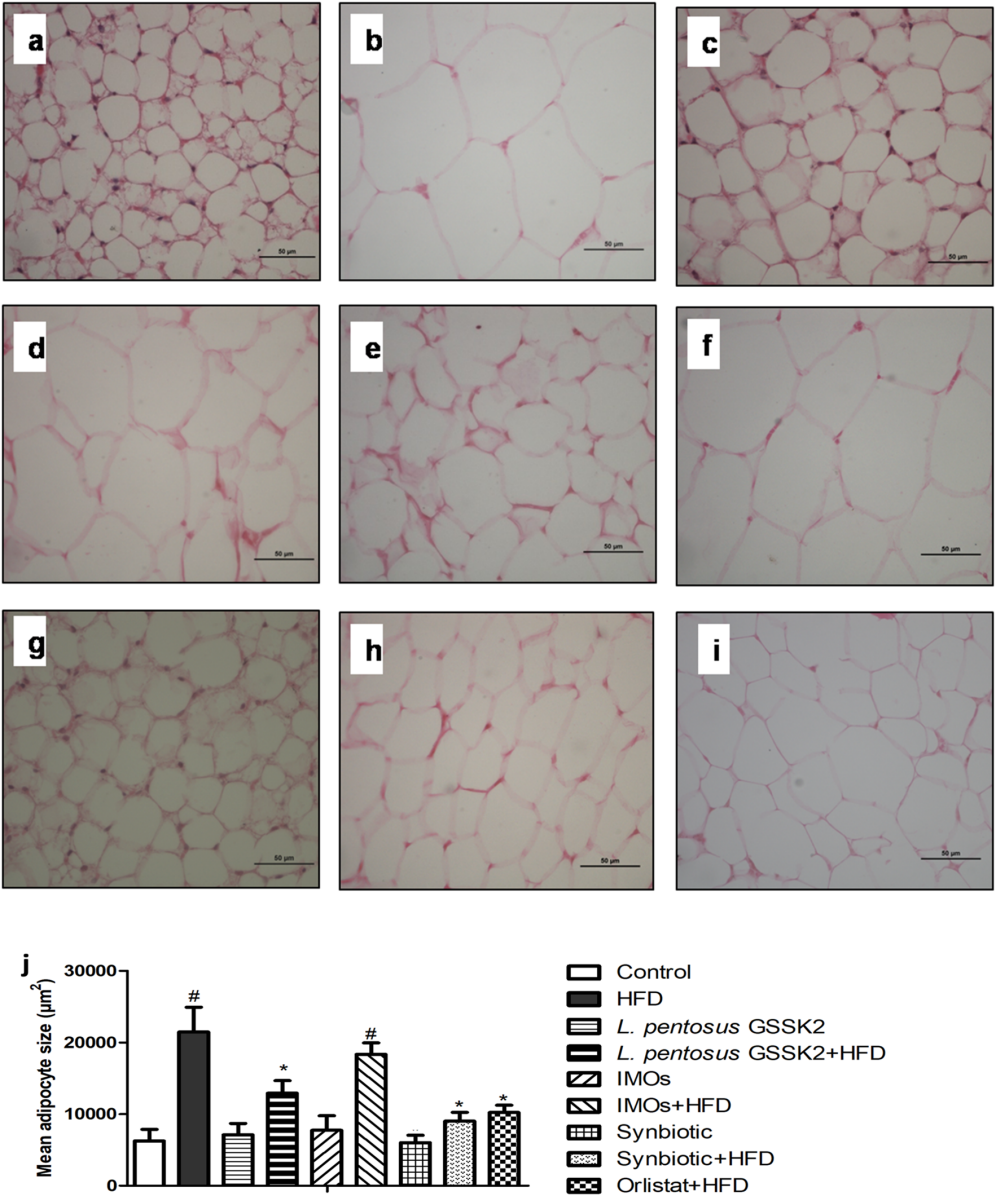
Photomicrograph of adipose tissue showing: (**a**) normal histomorphology with uniform, spherical adipocytes in control animals; (**b**) hypertrophied adipocytes in HFD; (**c**,**e**,**g**) normal histoarchitecture of adipocytes in *L. pentosus*, IMOs and synbiotic animals; (**d**,**f**,**h**,**i**) reduced adiposity in *L. pentosus* + HFD, IMOs + HFD, synbiotic + HFD and orlistat + HFD animals (H & E staining; scale bar: 50 µm, 400X); (**j**) mean adipocyte size in animals belonging to different groups.

Histological analysis of adipose tissue of HFD (Group II) animals showed hypertrophied adipocytes indicated by increased mean adipocyte size compared with normal histoarchitecture of adipocytes of control (Group I) animals (Fig. 5a,b,j). Interestingly, supplementation of probiotic, prebiotic, synbiotic and orlistat to HFD animals for 12 weeks led to reduced adipocyte hypertrophy. Maximum reduction in mean adipocyte size was observed in synbiotic + HFD (Group VIII) followed by orlistat + HFD (Group IX), *L. pentosus* GSSK2 + HFD (Group VI) and IMOs + HFD (Group VI) animals respectively (Fig. 5d,f,h–j). However, adipose tissue of animals belonging to either probiotic (Group III), prebiotic (Group V), or synbiotic (Group VII) had normal histoarchitecture of adipocytes (Fig. 5c,e,g).

**Figure 6:**
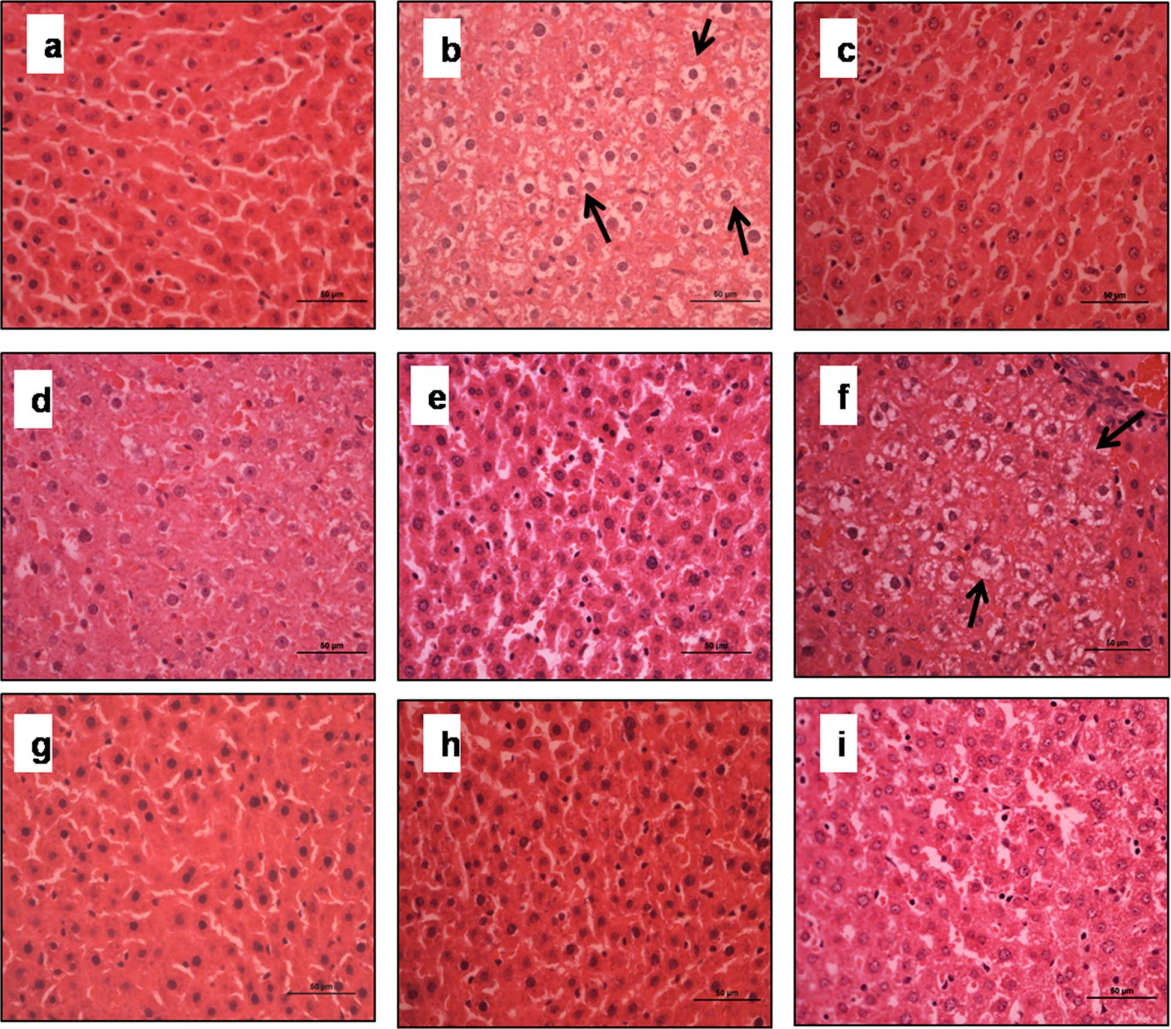
Photomicrograph of liver of animals belonging to different groups showing: (**a**) Normal histomorphology with polyhedral hepatocytes having large, rounded vesicular nuclei in control; (**b**) severe hepatic steatosis and ballooning degeneration of hepatocytes in HFD; (**c**,**e**,**g**) normal histoarchitectureof hepatocytes in *L. pentosus*, IMOs and synbiotic animals; (**f**) ballooned hepatocytes with vacuolated nuclei in IMOs + HFD animals; (**d**,**h**,**i**) reduced hepatic steatosis in *L. pentosus* + HFD, synbiotic + HFD and orlistat + HFD animals (H & E staining; arrows indicate ballooned hepatocytes; scale bar: 50 µm, 400X).

Morphological examination of liver of animals belonging to HFD (Group II) showed ballooning degeneration of hepatocytes and hypertrophy compared with normal hepatocyte of probiotic (Group III), prebiotic (Group V), synbiotic (Group VII) and control (Group I) animals (Fig. 6a,b). However, supplementation of either probiotic (Group IV), synbiotic (Group VIII) or orlistat (Group IX) to HFD animals led to reduced hepatic steatosis along with minimal fat deposition in hepatocytes compared with HFD (Group II) animals whereas, IMOs + HFD (Group VI) had increased hepatic steatosis with ballooned hepatocytes (Fig. 6c–i).

**Figure 7:**
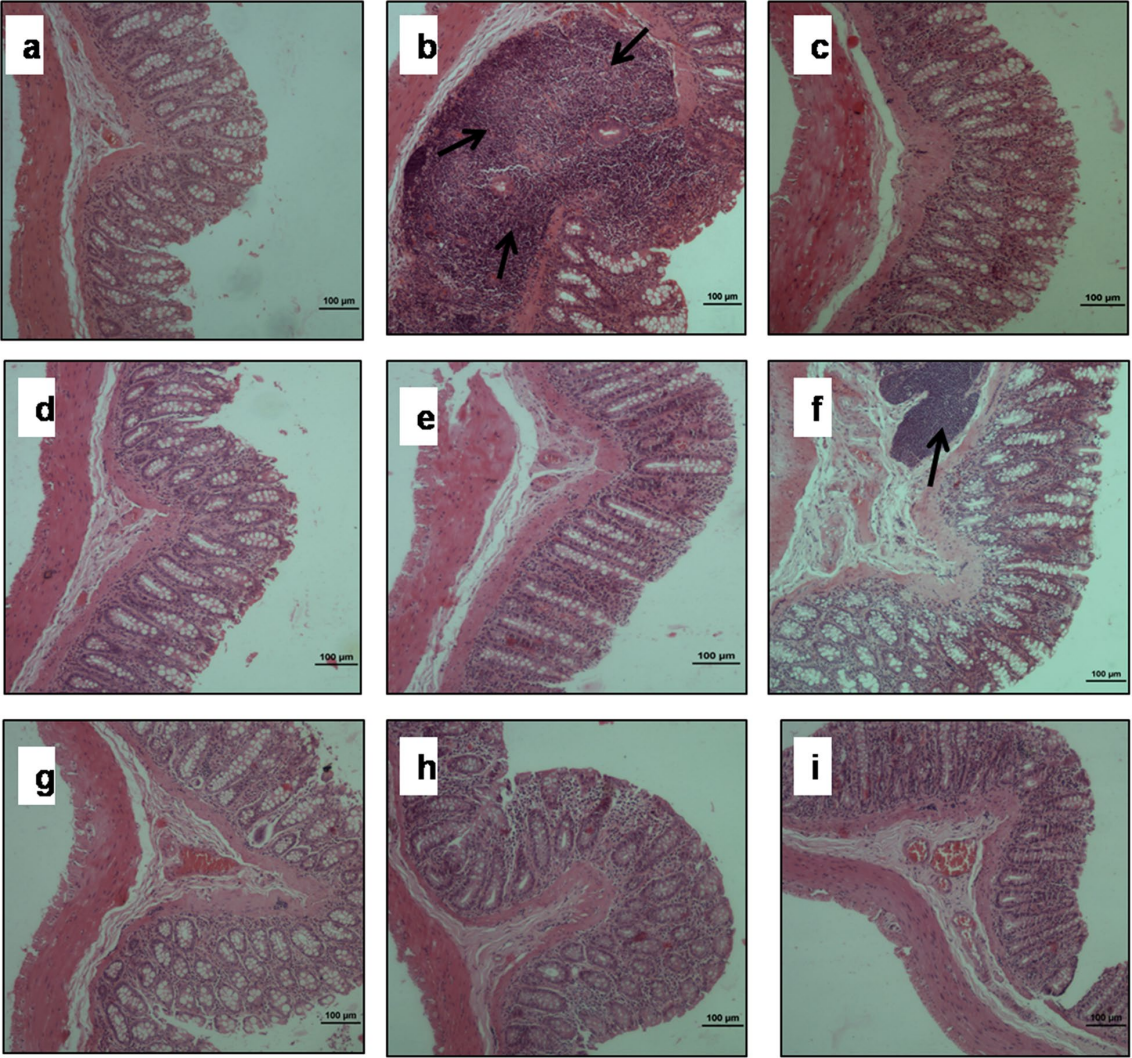
Photomicrograph of colon of animals belonging to different groups depicting: (**a**) normal histoarchitecture showing mucosa, submucosa, muscularis propria and serosa in control; (**b**) severely damaged mucosa with infilteration of lymphocytes and plasma cells in HFD; (**c**,**e**,**g**) normal histomorphology of colon in *L. pentosus*, IMOs and synbiotic animals; (**f**) inflammation in IMOs + HFD animals; (**d**,**h**,**i**) reduced infilteration of immune cells with intact mucosa in *L. pentosus* + HFD, synbiotic + HFD and orlistat + HFD animals (H & E staining; arrows indicate infilteration of inflammatory cells; scale bar: 100 µm, 100X).

Colon segments of HFD (Group II) animals showed disrupted crypts, focal colitis in form of excess lymphocytes between glands and hyperplasia compared with intact mucosal epithelium of probiotic (Group III), prebiotic (Group V), synbiotic (Group VII) and control (Group I) animals (Fig. 7a–c,e,g). Interestingly, the colon of *L. pentosus* GSSK2 + HFD (Group IV), synbiotic + HFD (Group VIII) and orlistat + HFD (Group IX) had intact epithelium lining and closely packed mucus glands with minimal inflammatory cells compared with accumulation of lymphocytes in colonic mucosa of IMOs + HFD (Group VI) animals (Fig. 7d,f,h,i).

## Discussion

Trialogue between diet, gut microbiota and host immune response has revealed novel prophylactic interventions employing probiotics for metabolic diseases. In our earlier studies, we have observed that indigenous probiotic *L. pentosus* GSSK2 showed potent anti-inflammatory activity in LPS induced RAW 264.7 cells, metabolized prebiotic IMOs and ameliorated adiposity parameters in HFD fed SD rats^14,15^. In present study, an attempt was made to investigate the prophylactic potential of a novel synbiotic intervention (*L. pentosus* GSSK2 + IMOs) in experimental metabolic syndrome compared with orlistat, commonly used weight loss drug.

Increased body weight is an important hallmark of metabolic syndrome and is accompanied by fat mass deposition. Interestingly, synbiotic + HFD animals had improved adiposity parameters with no change in average feed intake which might be attributed to lipoprotein lipase inhibition and release of appetite-reducing hormones glucagon-like peptide-1 and peptide YY either by probiotic or its metabolites resulting into increased energy expenditure, reduced visceral adipose tissue deposits and decreased mean adipocyte size^17–19^. Esposito et al.^20^ have also observed that supplementation of VSL#3 to HFD rats reduced fat mass with equal food intake. Hyperglycemia, a key outcome of metabolic syndrome, was ameliorated in synbiotic + HFD animals along with reduced insulin level suggesting improved glucose metabolism which might be due to alleviated β cell dysfunction, increased expression of GLUT-4 leading to increased glucose uptake by adipose tissue and muscles^21–23^. Lim et al. have also demonstrated that probiotic *Latilactobacillus sakei* OK67 reduced the blood glucose levels in HFD-fed mice due to decrease in LPS producing Gram negative bacteria and preventing LPS induced inflammation^24^.

Accumulating evidences have demonstrated the role of gut dysbiosis in the etiology and progression of diet induced disorders^25^. The decreased ratio of Bacteroidetes to Firmicutes and decreased abundance of beneficial bacteria i.e. *Bifidobacteria spp., Akkermansia spp.* along with increased pathobionts i.e. *Enterobacteriaceae* in HFD animals compared with control is consistent with earlier reports and are considered as “dysbiotic signatures”^13,26^. This dysbiotic microbiota contributes to metabolic diseases by increasing energy harvest which could be due to downregulation of angiopoietin-like protein 4, inducing host adiposity^2,27^. Further, increased abundance of LPS-producing Gram negative bacteria leads to elevated serum LPS thereby rupturing the gut barrier integrity, causing TLR-4 induced inflammation and endotoxemia^6,28^. Interestingly, in present study, increased Bacteroidetes to Firmicutes ratio, increased population of *Lactobacillus spp., Akkermansia spp., Faecalibacterium spp., Roseburia spp.* and decreased abundance of *Enterobacteriaceae* in feces of synbiotic + HFD animals along with increased weekly LAB count confirmed the shift from obesogenic to non-obesogenic bacteria and is in agreement with previous studies where similar results were observed on administration of probiotic *Latilactobacillus sakei* CJLS03 and synbiotic (*Lacticaseibacillus paracasei* HII01 + xylooligosaccharides) to HFD animals^29,30^. These beneficial bacteria i.e. *Lactobacillus spp., Faecalibacterium spp., Akkermansia spp*., and *Roseburia spp.* might have metabolized non-digestible complex carbohydrates such as resistant starch, bran present in diet, IMOs present in synbiotic, leading to short chain fatty acid production, especially butyrate, acetate and propionate via butyryl-CoA:acetate CoA-transferase pathway, propanediol pathway, succinate pathway etc. thereby regulating glucose and energy metabolism, promoting satiety, reducing systemic LPS levels, gut permeability thus preventing metabolic endotoxemia^31–34^.

It was observed that, synbiotic supplementation to HFD animals positively modulated the serum lipid profile paralleled with reduced hepatic steatosis in histological analysis and increased fecal lipid excretion thereby suggesting that synbiotic actually interfered with dietary lipids, making them indigestible and promoting their excretion instead of redistributing to the liver. Similarly, Bao et al. have also observed improved lipid profile on the administration of *Lactiplantibacillus plantarum* P‐8 to hyperlipidemic rats which may be due to increased bile salt hydrolase activity, cholesterol binding and assimilation by the probiotic cell walls or physiological actions of the metabolites produced^35^. The improved liver biomarkers (ALT, AST and serum bilirubin) in synbiotic + HFD animals is in concordance with earlier study where scientists have also observed reduced serum ALT and AST levels upon administration of a probiotic mixture (*Lactobacillus* and *Bifidobacterium*) to HFD-fed rats^36^. Further, enhanced antioxidants (GSH and SOD) and reduced oxidant (MDA) in synbiotic + HFD animals might be due to quenching of free radicals by probiotic leading to regulation of host redox status i.e. downregulating enzyme producing reactive oxygen species and increased antioxidant metabolites^37,38^. Li et al. have also demonstrated amelioration of liver oxidative stress i.e. increased SOD, GSH and reduced MDA in HFD-mice supplemented with *L. plantarum* strains^26^.

On molecular basis, synbiotic supplementation to HFD animals downregulated the expression of lipid metabolism regulators, FASN and HSL leading to suppressed lipid synthesis, increase β-oxidation, improved hepatosteatosis and corroborates with earlier studies where selenium enriched probiotics reduced the expression of FASN while Singh et al. reported decreased HSL expression in cobiotic (IMOs + lycopene) supplemented HFD animals^39,40^. Further, the liver GLUT-4 mRNA level was enhanced while glucokinase was reduced in synbiotic + HFD animals that might have attributed to improved insulin resistance and maintained glucose homeostasis^41^.

Interestingly, the expression of PPARγ and C/EBPα, the main transcription factors of adipocyte differentiation, lipid storage and adipokine signalling was reduced in synbiotic + HFD animals, suggesting decreased adipogenesis via limiting the conversion of preadipocytes to mature adipocytes^42^. Park et al. have also reported that *L. plantarum* Q180 inhibited 3T3-L1 adipocyte differentiation by downregulation of C/EBPα and PPARγ and reduction of adipocyte size in diet-induced obese mice^43^. Obesity is often accompanied by resistance to leptin, a hormone secreted by adipocytes, leading to increased hunger and reduced energy expenditure occurring due to hyperleptinemia^44^. Notably, synbiotic supplementation to HFD animals led to decreased leptin mRNA expression and is in agreement with previous study where *L. plantarum* A29 supplementation reduced fat mass and downregulated the expression of leptin in adipocytes, resulting in reduced bodyweight of HFD mice^45^. It was also noted that synbiotic + HFD animals had decreased expression of proinflammatory markers, TNF-α and IL-6, upregulated expression of anti-inflammatory adipokine i.e. adiponectin in adipose tissue along with reduced serum level of inflammatory markers (LPS, TNF-α and IL-6) which corroborated with previous study where administration of *L. pentosus* S-PT84 to LPS and HFD fed mice exerted anti-inflammatory effect by restoring adiponectin production and decreased pro-inflammatory mediators^46,47^. Alleviation of systemic endotoxemia by synbiotic intervention could be due to decreased LPS producing *Enterobacteriaceae* in the gut and downregulation of key signaling pathways by probiotic or its metabolites as observed in our earlier in vitro study, where *L. pentosus* GSSK2 attenuated LPS-induced inflammation by downregulating MAPK pathway and COX-2^14,48,49^.

In the present study, mucin gene Muc-2 and tight junction protein claudin were upregulated with reduced expression of TLR-4 in colon suggesting that synbiotic supplementation to HFD animals attenuated mucosal damage and regulated gut barrier function which is further supported by reduced infilteration of immune cells in colon of synbiotic + HFD animals. Similarly, Mennigen et al. reported that probiotic mixture VSL#3 protected the epithelial barrier function by maintaining tight junction expression in murine colitis model^50^.

Based on the present study, synbiotic biointervention (*L. pentosus* GSSK2 + IMOs) was found to be the most effective and comparable to antiobesity drug orlistat in terms of improved anthropometric parameters, biochemical markers, gene expression and histoarchitecture of metabolic tissues. Thus, the results clearly highlight that the protective potential of the synbiotic biointervention is multifarious and is mediated via its modulation of adipocytes, liver, colon and immune cells as all these play critical role in regulating metabolic homeostasis. Therefore, the proposed molecular mechanism of modulation of HFD-induced metabolic alterations by synbiotic may be attributed to remodulation of gut microbiota by probiotic as well as prebiotic, that may have led to altered adiposity by remodeling energy metabolism, activation of nutrient sensing pathways, mobilizing fats by regulating the expression of glucose and lipid metabolism genes, increased fatty acid oxidation, cholesterol binding and assimilation by probiotic. Moreover, increased short chain fatty acid production due to biofermentation of prebiotic and reduced circulating LPS levels due to decreased pathobionts may have led to mitigation of chronic inflammation, metabolic endotoxemia and restoration of intestinal barrier function that in turn regulated the glucose homeostasis and prevented fat accumulation in liver and adipose tissue thereby attenuating the progression of metabolic syndrome.

However, fecal microbiota analysis by next generation sequencing and short chain fatty acid analysis would have provided a better understanding of the impact of synbiotic intervention on gut microbiota and metabolites produced while insulin tolerance test would have given better insight about alleviation of insulin resistance. Moreover, due to species and strain specific response of probiotics and entirely different gut microbiota of rodent, detailed translational safety and efficacy studies for substantiating the functional benefits of the synbiotic, standardizing optimum dosage and monitoring the variability in response to these intervention is required in human subjects. Taken together, it is proposed that such novel synbiotic intervention may be employed for development of functional foods to combat the growing incidence of metabolic syndrome that could be considered as a promising live bacteriotherapy for maintaining the metabolic homeostasis.

## Methods

### Animals

Male Sprague–Dawley (SD) rats (150-180 g) were used in the study as they are more vulnerable than the females to the impacts of HFD on metabolic alterations and were procured from inbred population of the Central Animal House, Panjab University, Chandigarh, India^51^. Rats were housed in polypropylene cages with a hygienic bed of husk in room with 12 h light/dark cycle, acclimatized for 7–10 days and given standard pellet diet and water ad libitum*.*

### Ethics declaration

All protocols related to the sampling, care and management of animals were approved by Institutional Animals Ethical Committee (IAEC), Panjab University, Chandigarh and the Committee for the Purpose of Control and Supervision on Experiments on Animals (PU/45/99/CPCSEA/IAEC/2017/27). All experiments were performed in accordance with Institutional guidelines and regulations. The study is reported in accordance with ARRIVE guidelines.

### Preparation of HFD

Standard pellet diet (SPD) (6% calories from fat) was procured from Ashirwad Industries, Chandigarh, India and HFD (60% calories from fat) was prepared in-house as described previously^15^.

### Preparation of dose

- **Probiotic:** 18 h old culture of indigenous probiotic *L. pentosus* GSSK2 was centrifuged at 4,000 × *g* for 10 min at 4 °C, washed, and suspended in phosphate buffer saline (PBS pH 7.4) to contain 1 × 10^9^ lactobacilli/0.1 ml^15^.
- **Prebiotic:** IMOs (1 g/kg body weight/ 0.1 mL PBS^40^) was used as prebiotic.
- **Synbiotic:** Probiotic *L. pentosus* GSSK2 (1 × 10^9^ lactobacilli/0.1 ml) in combination with prebiotic IMOs (1 g/kg body weight), was employed as synbiotic.

### Experimental design

Animals were divided into nine groups, each comprising of 6 animals and treated as follows.

- **Group I (Control):** Animals were fed with SPD for 12 weeks.
- **Group II (HFD):** Animals were fed with HFD for 12 weeks.
- **Group III (*L. pentosus* GSSK2):** Animals were fed with a single dose of probiotic (1 × 10^9^ lactobacilli/0.1 mL) daily via orogastric gavage and were given SPD for 12 weeks.
- **Group IV (*L. pentosus* GSSK2 + HFD):** Animals were fed orally with a single dose of probiotic (1 × 10^9^ lactobacilli/0.1 mL) daily along with HFD for 12 weeks.
- **Group V (IMOs):** Animals belonging to this group were fed orally with a single dose of IMOs (1 g/kg body weight) daily along with SPD for 12 weeks.
- **Group VI (IMOs + HFD):** Animals were fed orally with a single dose of IMOs (1 g/kg body weight/ 0.1 mL) daily along with HFD for 12 weeks.
- **Group VII (Synbiotic):** Animals were fed orally with a single dose of both probiotic (1 × 10^9^ lactobacilli/0.1 mL) and IMOs (1 g/kg body weight) daily along with SPD for 12 weeks.
- **Group VIII (Synbiotic + HFD):** Animals were fed orally with a single dose of both probiotic (1 × 10^9^ lactobacilli/0.1 mL) and IMOs (1 g/kg body weight) daily along with HFD for 12 weeks.
- **Group IX (Orlistat + HFD):** Animals were fed orally with a single dose of orlistat (10 mg/kg body weight/ 0.1 mL PBS) daily along with HFD for 12 weeks.

### Follow up of animals

Body weight and LAB count were monitored once a week, throughout the experiment. A day before sacrificing the animals, fasting blood glucose level was monitored, oral glucose tolerance test (OGTT) was performed and feces of animals were collected, for estimation of fecal lipids and analysis of gut bacterial abundance. Animals were sacrificed after 12 weeks of respective treatments by injecting ketamine hydrochloride (80 mg/kg) intraperitoneally followed by cervical dislocation. Blood was drawn through retro-orbital bleeding for estimation of serum biochemical parameters. Liver, adipose tissue (epididymal and retroperitoneal) and colon were collected for analysis of oxidants and antioxidants, histopathological alterations and molecular markers (Supplementary Fig. S2).

### Evaluation of morphometric parameters and adiposity markers

Body weight of animals was recorded weekly on ordinary balance (SD-300, S.D fine chemicals Ltd, Chandigarh, India) while abdominal circumference was measured at the beginning and end of the study using ordinary measuring tape^15^. Lee’s Index was calculated as cube root of body weight (g)/ naso-anal length (cm), Body mass index (BMI) was monitored as body weight (g)/length^2^ (cm^2^) at the end of experiment^13^.

Feed intake of animals was recorded twice a month and was calculated by subtracting the amount of residual food in each cage from the weighed amount of food provided on previous day (g/day) and represented as average feed intake (g/day/ rat) by dividing the feed intake by total number of animals per cage^15^. Post sacrifice, liver and adipose tissue were weighed using ordinary balance.

### Blood glucose and OGTT

Fasting blood glucose levels of animals were recorded weekly via tail snip method using glucometer (Freestyle Optium Glucometer, Abbott Ltd., Oxon, UK). For OGTT, animals were fasted for 6 h and blood glucose level was measured before and after oral administration of D-glucose (2 g/kg) at an interval of 15, 30, 60, 90, and 120 min respectively^15^. AUC was calculated using GraphPad PRISM 5 software. Serum insulin level was determined using commercially available ELISA kit as per manufacturer’s instructions (Ray Biotech, Norcross, GA, USA).

### Fecal LAB count and fecal lipids

To assess the effect of synbiotic supplementation on LAB in the colon, freshly voided fecal material (0.5 g/animal) was collected weekly from each group, homogenized in normal saline, serially diluted and plated on MRS agar. The plates were incubated at 37 °C for 48 h and colony forming units (CFU) were recorded^11^.

Fecal lipids were extracted using phase separation based method by Folch et al.^52^ followed by estimation of total lipids by method of Fringes and Dunn^53^. Briefly, 200 mg dry feces was taken in a centrifuge tube and 3 mL of chloroform–methanol mixture (2:1, v/v) was added, vortexed for 1 min and centrifuged at 3,000 g for 10 min. The chloroform phase containing lipid fraction was collected in a fresh tube and completely dried followed by estimation of total lipids.

### Selected gut bacterial abundance

Bacterial DNA was isolated using QIAmp® DNA stool mini kit (Qiagen, Hilden, Germany) from 180 mg of fecal sample, as per manufacturer’s instruction. DNA quantification was performed using Infinite® M200 Pro NanoQuant (Tecan). DNA extracted as above was subjected to qPCR to quantify the abundance of *Lactobacillus spp., Bifidobacterium spp., Roseburia spp., Akkermansia spp., Faecalibacterium spp., Ruminococcus spp., Prevotella spp.,* using genus-specific primers and that of *Enterobacteriaceae*, Bacteroidetes and Firmicutes using phylum-specific primers taking total bacteria as an internal control. q-PCR conditions and primer details are given in supplementary material (Supplementary Table 1). Data was analyzed using the ΔΔCt method and values expressed as fold change relative to the control group^13^.

### Analysis of serum biochemical parameters

Blood was collected retro-orbitally and serum was prepared to estimate liver function test [Bilirubin, aspartate transaminase (AST) and alanine transaminase (ALT)] and lipid profile [Total cholesterol, triglycerides, high-density lipoprotein (HDL) cholesterol and low-density lipoprotein (LDL) cholesterol] using autoanalyser, Sysmex XP-100. Serum lipopolysaccharide (LPS), TNF-α and IL-6 were quantified using commercially available ELISA kits (BT Laboratory, Zhejiang, China) as per manufacturers’ instructions.

### Assessment of oxidant and antioxidant level

Tissue homogenates of colon, liver and adipose tissue samples were prepared in 0.15 M PBS (pH 7.2) using potter Elvehjem homogenizer. Post mitochondrial supernatant (PMS) was prepared by cold centrifuging tissues homogenates at 16,000 × g for 10 min and supernatant was labeled as PMS. Protein concentration in tissue homogenate and PMS was measured using standard method of Lowry et al.^54^.

The amount of malondialdehyde (MDA), a measure of lipid peroxidation, was assayed in homogenates as per Wills^55^ and results were expressed as nanomoles of MDA per milligram of protein. Superoxide dismutase (SOD) activity was assayed in PMS of tissue homogenates according to the method of Kono^56^ and expressed as units of SOD per milligram of protein, where 1 U activity is defined as the amount of SOD required to inhibit the rate of Nitroblue tetrazolium reduction by 50%. Reduced glutathione (GSH) levels were estimated in tissue homogenates as per Ellman^57^, absorbance was measured at 412 nm and results were expressed as µmole of GSH/mg of protein.

### Gene expression analysis

q-PCR based gene expression analysis was done for fatty acid synthase (FASN), hormone sensitive lipase (HSL), glucokinase, GLUT-4, TNF-α and IL-6 in liver; CCAAT/ enhancer- binding protein alpha (C/EBPα), peroxisome proliferators-activated receptor gamma (PPAR-γ), leptin, adiponectin, TNF-α and IL-6 in adipose tissue; claudin, CDX-2, Muc2 and TLR-4 in colon. Total RNA was extracted using Trizol (Sigma Aldrich, USA). 1 µg of RNA sample was used for c-DNA synthesis using commercially available kit (Biorad iscript kit 1,708,891) as per the kit’s instructions. Relative expression of different genes was determined by qPCR using SYBR® based dye (Biorad C1000 Touch Real-Time PCR machine). q-PCR conditions and primer details are given in supplementary material (Supplementary Table 2). Data was analyzed using the ΔΔCt method and values were expressed as fold change relative to control group. GAPDH was used as internal reference gene to normalize the expression of target genes.

### Histological analysis

A part of distal colon, liver and adipose tissue were fixed immediately in 10% buffered formalin, processed, stained with hematoxylin and eosin, and examined for histological alterations using light microscope. The mean adipocyte sizes in adipose tissue sections (minimum 2 animals per group) were estimated in 10–12 images (40X objective), using Image J software^58^.

### Statistical analysis

Results were expressed as mean ± standard deviation (SD). The inter group variation was assessed by one-way analysis of variance (ANOVA) followed by Tukey’s post Hoc Test using PRISM software (5.0). The statistical significance was defined as p and calculated at p < 0.05.

## Supplementary Information

Supplementary Information.
