# Supplementary material for: Synbiotic (Lactiplantibacillus pentosus GSSK2 and isomalto-oligosaccharides) supplementation modulates pathophysiology and gut dysbiosis in experimental metabolic syndrome
Source: Sci Rep. 2021 Nov 1;11:21397. doi: 10.1038/s41598-021-00601-2 (PMC8560755; doi:10.1038/s41598-021-00601-2)
Supplement: Supplementary file 1 — Supplementary Information. [file 41598_2021_601_MOESM1_ESM.pdf]

Synbiotic (*Lactiplantibacillus pentosus* GSSK2 and isomalto-oligosaccharides) supplementation modulates pathophysiology and gut dysbiosis in experimental metabolic syndrome

Sakshi Khanna[1], Mahendra Bishnoi[2], Kanthi Kiran Kondepudi[2*], Geeta Shukla[1*]

[1]Department of Microbiology, Panjab University, Chandigarh, India

[2]Healthy Gut Research Group, National Agri-Food Biotechnology Institute (NABI), S.A.S. Nagar, Punjab, India

[*]Corresponding author

Professor Geeta Shukla

Department of Microbiology, Basic Medical Sciences Block A, South Campus, Panjab University, Chandigarh, 160014, India. Email:geeta_shukla@pu.ac.in

ORCID: 0000000212122211

Dr. Kanthi Kiran Kondepudi

Healthy Gut Research Group, Food & Nutrition Biotechnology Division, National Agri-Food Biotechnology Institute (NABI), S.A.S. Nagar, Punjab 140306, India. Email: kiran@nabi.res.in

Supplementary Information

Supplementary Table S1: List of bacterial primers for q-PCR analysis of gut bacterial abundance

Bacterial primers	Forward 5'-3'	Reverse 5'- 3'
Total Bacteria	ACTCCTACGGGAGGCAGCAGT	ATTACCGCGGCTGCTGGC
Bacteroidetes	ACGCTAGCTACAGGCTTAACA	ACGCTACTTGGCTGGTTCA
Firmicutes	GCGTGAGTGAAGAAGT	CTACGCTCCCTTTACAC
Lactobacillus	CACCGCTACACATGGAG	AGCAGTAGGGAATCTTCCA
Bifidobacterium	TCGCGTCYGGTGTGAAAG	CCACATCCAGCRTCCAC
Akkermensia	CAGCACGTGAAGGTGGGGAC	CCTTGCGGTTGGCTTCAGAT
Faecalibacterium	GAGGAAGATAATGACGGTAC	ACCTCTGCACTACTCAAGA
Roseburia	GCGGTRCGGCAAGTCTGA	CCTCCGACACTCTAGTMCGA
Ruminococcus	CCCTGCGCTGTGCGAAAAAG	CCGCTGCGTACCTTTGGGAT
Prevotella	GATGGGGATGCGTCTGATTAG	TCCTGCACGCTACTTGGCT
Enterobacteriaceae	CCCTTATTGTTAGTTGCCAT	ACTCGTTGTACTTCCCATTG

Primer sequences were obtained from literature[13] and were procured from Eurofin Genomics India Pvt. Ltd.

PCR conditions for gut bacteria abundance : q-PCR was performed in 10µl reaction volume containing 1 µl template DNA (40 ng), 1 µl forward primer, 1 µl reverse primer, 2µl RNase free water and 5 µl iTaq Universal SYBR Green Supermix (2X) and was carried out using following conditions: 95°C for 2 minutes followed by 40 cycles of 95°C for 5 s, 60°C for 30 s with melt curve at 65-95°C for 5 s.

Supplementary Table S2: List of primers for q-PCR analysis of gene expression in tissues.

Gene	Forward (5'-3')	Reverse (5'-3')	Source
**GAPDH**	ACGGGAAACCCATCACCATC	CTCGTGGTTCACACCCATCA	*
**Adiponectin**	GGAAACTTGTGCAGGTTGGATG	GGGTCACCCTTAGGACCAAGAA	[1]
**Leptin**	TTCAAGCTGTGCCTATCCACAAAG	TGAAGCCCGGGAATGAAGTC	[1]
**TLR-4**	TGGCAGTTTCTGAGTAGCCG	GCTTTTCCATCCAACAGGGC	[2]
**Claudin**	TGTCCACCATTGGCATGAAG	GCCACTAATGTCGCCAGACC	[3]
**CDX-2**	CGCATCATCACCCTCACCAT	CGTCCTGGTTTTCACTTGGC	*
**Muc-2**	GCCAGATCCCGAAACCATGT	AGGACGGACTCTATGCTGGA	[2]
**GLUT-4**	CAACTGGACCTGTAACTTCATCGT	ACGGCAAATAGAAGGAAGACGTA	[4]
**Glucokinase**	CAGTGGAGCGTGAAGACAAA	CTTGGTCCAATTG AGGAGGA	*
**CEBP-α**	TTACAACAGGCCAGGTTTCC	GGCTGGCGACATACAGTACA	[5]
**PPAR-γ**	GACCACTCCCATTCCTTTGA	CGCACTTTGGTATTCTTGGAG	[6]
**IL-6**	TCCTACCCCAACTTCCAATGCTC	TTGGATGGTCTTGGTCCTTAGCC	[7]
**TNF-α**	GTCGTAGCAAACCACCAAGC	TGTGGGTGAGGAGCACATAG	[8]
**FASN**	GCAGCAGCATGATGTAGCAC	AGTTGCACACCACAAGGTCA	*
**HSL**	CGCCTTACGGAGTCTATGC	TCTGATGGCTCTGAGTTGC	[5]

*Primer sequences were designed using Primer BLAST software and were procured from Eurofin Genomics India Pvt. Ltd.

PCR conditions for gene expression analysis in tissues: q-PCR was performed in 10µl reaction volume containing 1 µl template cDNA (20 ng), 1 µl forward primer, 1 µl reverse primer,1 µl RNase free water and 5 µl iTaq Universal SYBR Green Supermix (2X). qPCR was carried out under the following conditions: 95°C for 2 minutes followed by 40 cycles of 95°C for 5 s, 60°C for 30 s with melt curve at 65-95°C for 5 s.

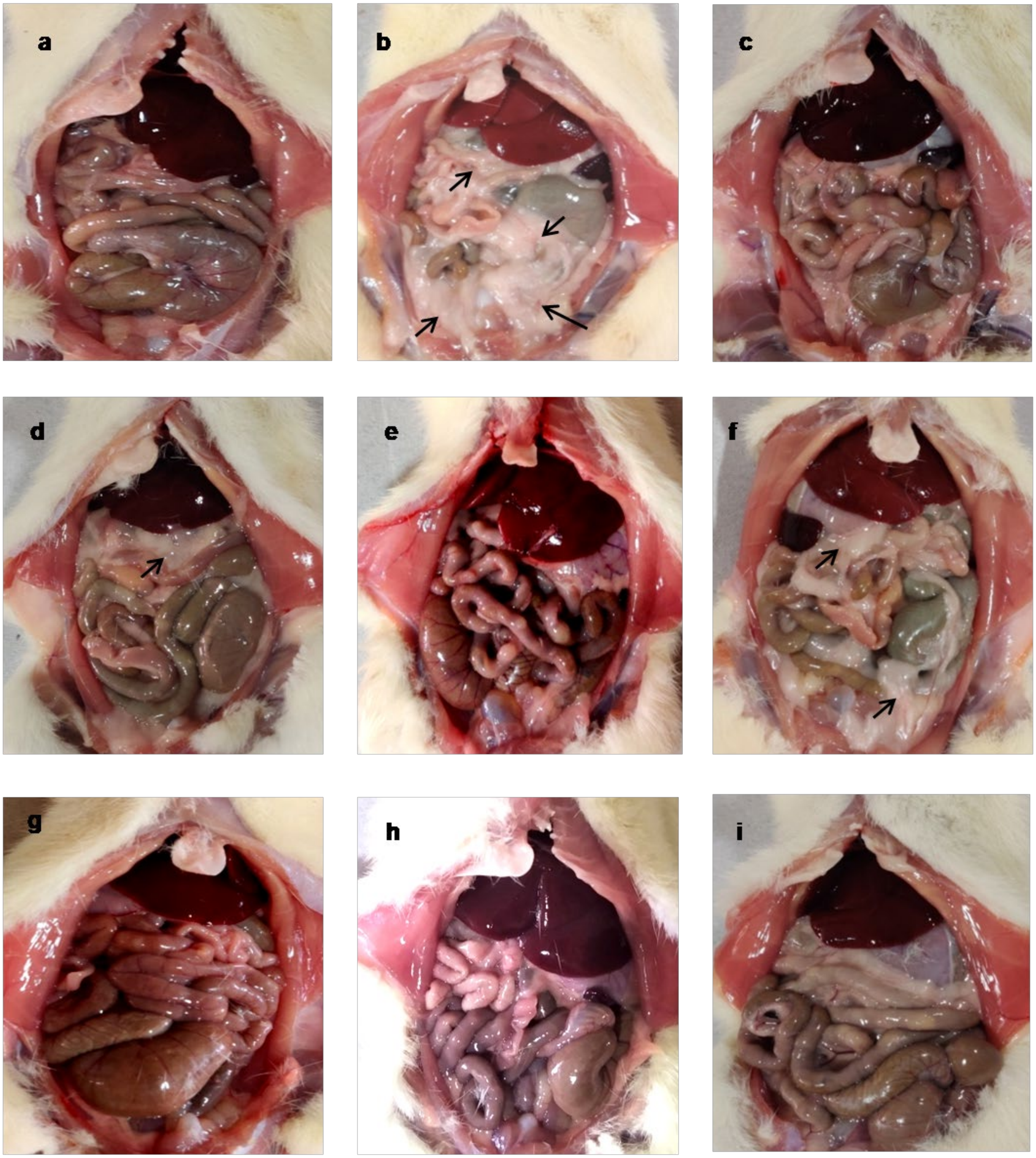

Figure S1: Gross macroscopic observation of adipose tissue of animals belonging to different groups: (a) Control, (b) HFD, (c) *L. pentosus*, (d) *L. pentosus* + HFD, (e) IMOs, (f) IMOs + HFD, (g) Synbiotic, (h) Synbiotic + HFD, (i) Orlistat + HFD. Arrows indicate adipose tissue deposits.

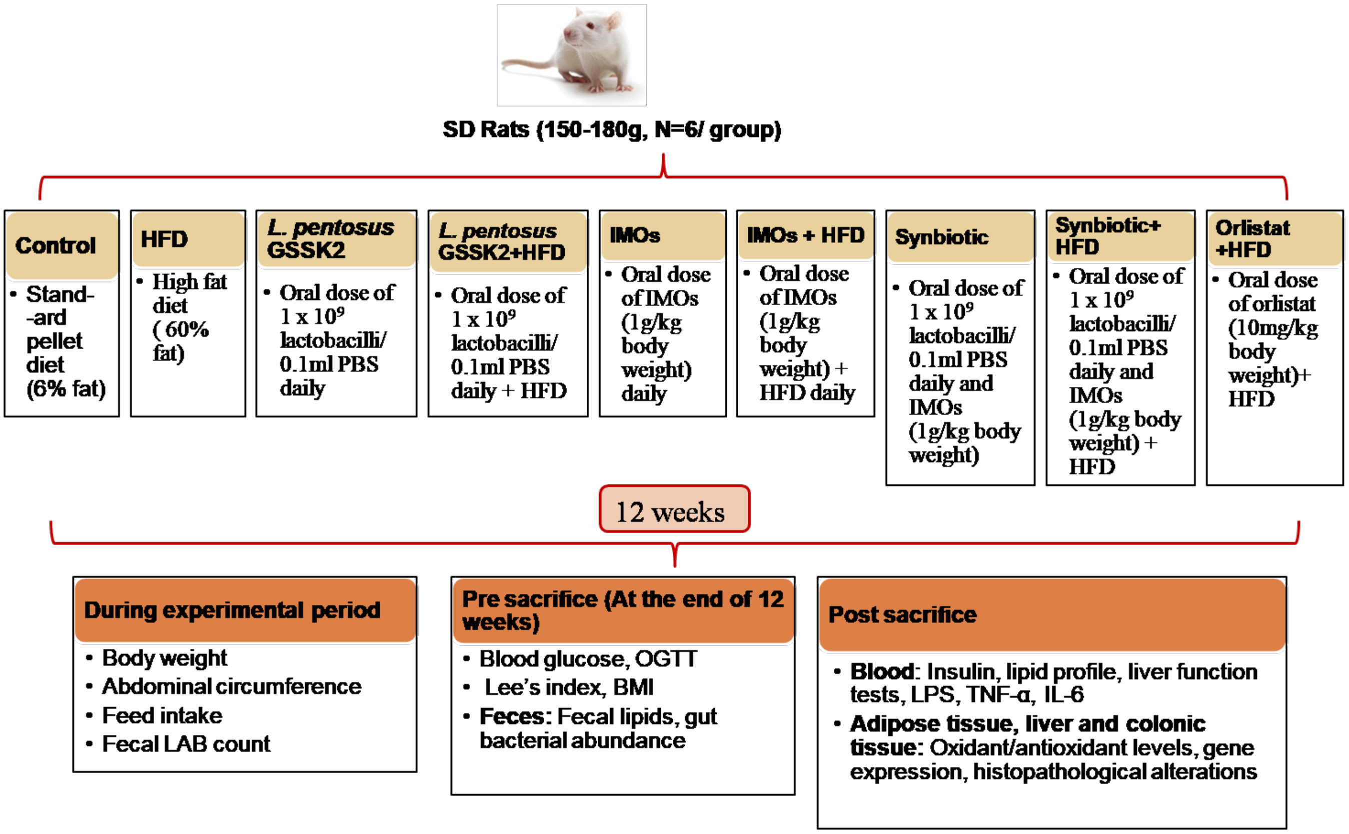

Figure S2: Flow diagram depicting different experimental groups and interventions.

References

1. Abd El-Haleim, E.A., Bahgat, A.K., Saleh, S. Resveratrol and fenofibrate ameliorate fructose-induced nonalcoholic steatohepatitis by modulation of genes expression. *World J Gastroenterol* **22**, 2931-2948 (2016)

2. Kumar, V., Mahajan, N., Khare, P., Kondepudi, K.K., Bishnoi, M. Role of TRPV1 in colonic mucin production and gut microbiota profile. *Eur J Pharmacol*. **888**, 173567; 10.1016/j.ejphar.2020.173567 (2020).

3. Takizawa, Y., Kishimoto, H., Kitazato, T., Tomita, M., Hayashi, M. Changes in protein and mRNA expression levels of claudin family after mucosal lesion by intestinal ischemia/reperfusion. *Int J Pharm* **426**, 82-89 (2012).

4. Qin, B., Polansky, M. M., Harry, D., Anderson, R. A. Green tea polyphenols improve cardiac muscle mRNA and protein levels of signal pathways related to insulin and lipid

metabolism and inflammation in insulin-resistant rats. *Mol Nutr Food Res*. **54**, S14-23. (2010)

5. Li, X. et al. Fructus xanthii improves lipid homeostasis in the epididymal adipose tissue of rats fed a high-fat diet. *Mol Med Rep* **13**, 787-795 (2016).

6. Alonso, M. et al. Anti-obesity efficacy of LH-21, a cannabinoid CB(1) receptor antagonist with poor brain penetration, in diet-induced obese rats. *Br J Pharmacol* **165**, 2274-2291 (2012).

7. Parafati, M. et al. Bergamot Polyphenols Boost Therapeutic Effects of the Diet on Non-Alcoholic Steatohepatitis (NASH) Induced by "Junk Food": Evidence for Anti-Inflammatory Activity. *Nutrients* **10**, 1604; 10.3390/nu10111604 (2018).

8. Agarwal, D., Dange, R. B., Vila, J., Otamendi, A. J., Francis, J. Detraining differentially preserved beneficial effects of exercise on hypertension: effects on blood pressure, cardiac function, brain inflammatory cytokines and oxidative stress. *PLoS One* **7**, e52569; 10.1371/journal.pone.0052569 (2012).
